# Nuclear lamina dysfunction triggers a germline stem cell checkpoint

**DOI:** 10.1038/s41467-018-06277-z

**Published:** 2018-09-27

**Authors:** Lacy J. Barton, Tingting Duan, Wenfan Ke, Amy Luttinger, Kaylee E. Lovander, Alexey A. Soshnev, Pamela K. Geyer

**Affiliations:** 1 Department of Biochemistry, University of Iowa, Iowa City, IA 52242 USA; 2 Present Address: Department of Cell Biology, Skirball Institute, NYU School of Medicine, 540 First Avenue, New York, NY 10016 USA; 3 Present Address: Department of Biology, University of Virginia, 485 McCormick Rd, Charlottesville, VA 22904 USA; 4 Present Address: Laboratory of Chromatin Biology and Epigenetics, The Rockefeller University, 1230 York Avenue, New York, NY 10065 USA

## Abstract

LEM domain (LEM-D) proteins are conserved components of the nuclear lamina (NL) that contribute to stem cell maintenance through poorly understood mechanisms. The Drosophila emerin homolog Otefin (Ote) is required for maintenance of germline stem cells (GSCs) and gametogenesis. Here, we show that *ote* mutants carry germ cell-specific changes in nuclear architecture that are linked to GSC loss. Strikingly, we found that both GSC death and gametogenesis are rescued by inactivation of the DNA damage response (DDR) kinases, ATR and Chk2. Whereas the germline checkpoint draws from components of the DDR pathway, genetic and cytological features of the GSC checkpoint differ from the canonical pathway. Instead, structural deformation of the NL correlates with checkpoint activation. Despite remarkably normal oogenesis, rescued oocytes do not support embryogenesis. Taken together, these data suggest that NL dysfunction caused by Otefin loss triggers a GSC-specific checkpoint that contributes to maintenance of gamete quality.

## Abstract (web summary)

Otefin is a nuclear lamina protein required for survival of Drosophila germ stem cells. Here the authors show that nuclear lamina dysfunction resulting from loss of Otefin activates a DNA damage-independent germ stem cell-specific checkpoint, mediated by the ATR and Chk2 kinases, which ensures that healthy gametes are passed on to the next generation.

## Introduction

The nuclear lamina (NL) is an extensive protein network comprised of lamins and hundreds of associated proteins. Among these are the conserved family of LEM Domain (LEM-D) proteins, named for the founding human members LAP2, emerin, and MAN1^1,2^. These proteins share a LEM-D that interacts with Barrier-to-Autointegration Factor (BAF), a conserved double-stranded DNA and histone-binding protein that promotes chromosome condensation important for nuclear assembly^3,4^. Interactions between LEM-D proteins and BAF tether chromatin to the nuclear periphery, organizing the genome for transcription, DNA replication, and repair^5–7^. In addition, LEM-D proteins interact with transcriptional regulators, including repressors and nuclear effectors of signaling cascades^1,2^. Although LEM-D proteins are globally expressed, mutations in *LEM-D* genes cause tissue-restricted diseases, termed laminopathies, that affect tissues such as skeletal muscle, skin, fat, and bone^2,8^. A unifying feature of laminopathies is the age-dependent worsening of disease phenotypes, leading to the suggestion that laminopathies result from defects in tissue homeostasis and a failure to maintain adult stem cell populations^9,10^. Despite the growing evidence that LEM-D proteins are required in multiple types of adult stem cells, it remains unclear how LEM-D proteins maintain healthy stem cell populations and promote tissue homeostasis.

**Fig. 1:**
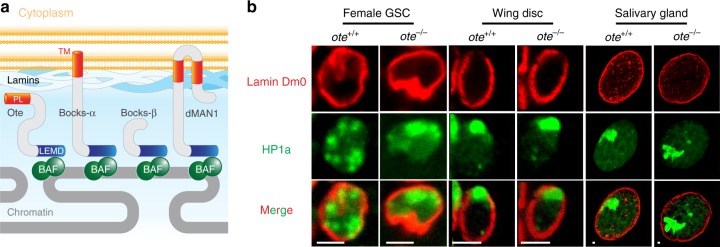
Loss of Ote causes germline stem cell-specific NL defects. **a** Schematic of the Drosophila nuclear lamina (NL). Underneath the inner nuclear envelope (yellow) resides the NL (blue) that contains three Drosophila LEM-D proteins, Otefin (Ote), Bocksbeutel (Bocks α and β), and dMAN1. Each protein localizes to the NL using a domain (orange, Peripheral localization, PL) that interacts with lamin or using transmembrane domains (orange, TM). Proteins in the LEM-D family carry a LEM-D (blue) that binds Barrier-to-Autointegration Factor (BAF, green), to promote chromatin assembly at the nuclear periphery. **b** Confocal images of *ote*^*+/+*^ and *ote*^*−/−*^ (*ote*^*B279G/PK*^) nuclei stained with antibodies against Lamin Dm0 (red) and Heterochromatin protein 1a (HP1a; green). Scale bars, 2.5 μm

*Drosophila melanogaster* has emerged as a powerful model to define the function of LEM-D proteins in tissue homeostasis. Drosophila encodes four LEM-D proteins^11^, of which three are NL-associated (Fig. 1a). These include Drosophila MAN1 (dMAN1) and two emerin homologs Otefin (Ote) and Bocksbeutel (Bocks). As in mammals, loss of individual Drosophila LEM-D proteins causes distinct tissue-restricted developmental defects that worsen with age, with little to no effect on viability^11–15^. Even so, LEM-D proteins have overlapping developmental functions, as loss of any two NL LEM-D proteins is lethal^13^. Studies of Ote revealed a key role for this LEM-D protein in stem cell homeostasis. Loss of Ote causes female and male sterility due to a failure in adult germline stem cell (GSC) maintenance^12,15,16^. As Drosophila GSC niches and germ cell properties are among the best characterized^17,18^, studies of Ote are instrumental for elucidating how LEM-D proteins support adult stem cells.

Loss of Ote blocks germ cell differentiation and causes progressive GSC death^12,16^. Here we investigate the mechanism behind these defects. We find that loss of Ote causes a thickening of the NL and reorganization of heterochromatin in GSC nuclei, but not in mutant somatic cell nuclei. Such architectural defects are reminiscent of phenotypes observed in human *LMNA* mutant progeroid (premature aging) models^19^, in which cellular senescence has been linked to compromised replication and DNA damage^20–23^. Prompted by these links, we investigated whether GSC death resulted from activation of a DNA damage response (DDR). We found that loss of Ataxia Telangiectasia and Rad3-related (ATR) or Checkpoint kinase 2 (Chk2) in *ote* mutants rescues oogenesis, with females laying eggs. Surprisingly, although rescue of oogenesis was achieved by loss of components in the DDR pathway, we demonstrate that canonical triggers are not responsible for pathway activation. Instead, we establish that Chk2 activation is linked to defects in NL structure. Rescued *chk2, ote* double-mutant oocytes do not support embryogenesis, suggesting that NL dysfunction in GSCs triggers a checkpoint pathway that maintains female gamete quality to safeguard offspring fitness. Our studies suggest that laminopathies might be linked to activation of similar quality control pathways that selectively impact specific adult stem cell populations.

## Results

### NL organization in GSCs is altered in the absence of Ote

Loss of Ote compromises GSC homeostasis in female and male gonads^12,16^. To define mechanisms responsible for these defects, we focused our studies on the ovary because *ote*^*−/−*^ mutant GSC phenotypes are immediately apparent in newborn females but have an age-dependent component in males^16^. Drosophila ovaries are divided into 16 to 20 ovarioles, which are arranged in advancing stages of oocyte maturation. At the anterior end of the ovariole is the germarium, a specialized structure that contains a stem cell niche that supports two to three GSCs (Supplementary Fig. 1A). Upon division, GSCs produce one daughter cell that remains attached to the somatic niche and receives niche factors critical for GSC self-renewal. The second daughter cell is displaced from the niche, does not receive self-renewal signals and differentiates. Germ cell differentiation begins with four incomplete mitotic divisions to generate a 16-cell cyst, which develops into an egg chamber containing 1 oocyte and 15 supporting nurse cells. In wild-type Drosophila, GSCs are maintained and gametes are produced throughout the lifespan of the animal, making this defined and tractable system instrumental to understanding how LEM-D proteins support adult stem cell function.

Ote localizes to the NL in somatic and germ cells, yet loss of Ote specifically affects germ cells. To understand whether this tissue-restricted requirement is linked to nuclear structure, we dissected ovaries from young (<1-day-old) females and stained them with antibodies against the B-type lamin (Lamin Dm0) and Heterochromatin Protein 1a (HP1a). Notably, germline progenitors and adult GSCs have a distinct nuclear organization from somatic cells. In GSCs, homologous chromosomes and centromeres are unpaired^24,25^, whereas in other cell types, homologous chromosomes are paired and centric regions cluster to form an HP1a-containing chromocenter^26^. We found that *ote*^*−/*^^−^ GSCs have a thick and irregular NL, as well as increased clustering of HP1a compared to wild-type GSCs (Fig. 1b). Strikingly, this altered nuclear organization is specific to *ote*^*−/−*^ GSCs because *ote*^*−/−*^ nuclei of imaginal disc tissues (2n) and salivary gland tissues (~1000n) are phenotypically indistinguishable from wild-type controls (Fig. 1b). Together, these findings imply that Ote loss is associated with germ cell-specific nuclear architectural defects.

### The DDR transducer kinase Chk2 causes *ote*^−^^*/−*^ GSC loss

**Fig. 2:**
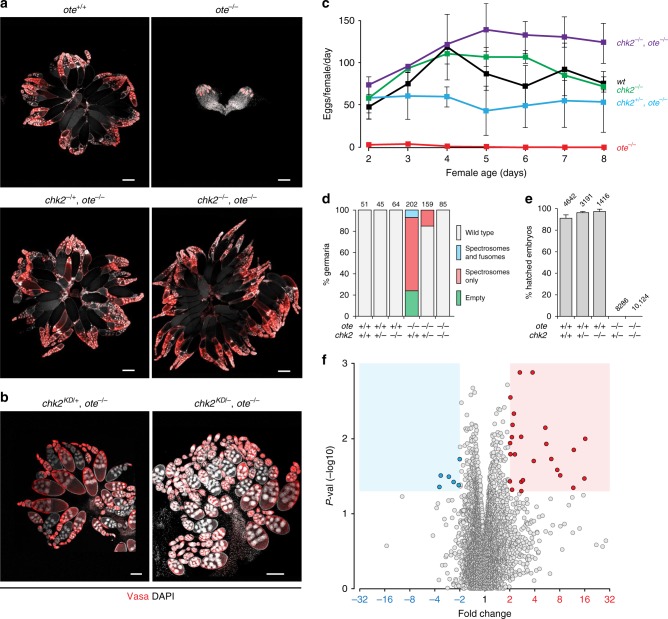
Chk2 activation is responsible for oogenesis defects in *ote* mutants. **a**, **b** Confocal images of ovaries dissected from less than 2-day-old females stained for Vasa (red) and DAPI (white). Genotypes are noted on the top of each image, wherein    *ote*^*−/−*^ corresponds to *ote*^*B279G/PK*^. The *chk2*^*-*^ alleles were either deletion alleles (**a**) or a kinase dead allele (Table S1, b). Scale bars represent 250 µm (**a**) or 100 µm (**b**). **c** Fecundity (eggs per female per day) of females mated to *ote*^*+/+*^ males as a function of female age in days. Bars indicate the standard deviation from a minimum of three independent experiments, assaying 7–15 females each time. Genotypes are noted to the right of each line. **d** Quantification of germarial phenotypic classes, as described on the right^12^. Genotypes are listed below the corresponding bar, with the number of germaria scored listed at the top. **e** Percentage of eggs that hatched within 48 h following deposition by females that were mated to *ote*^*+/+*^ males. Bars represent the standard deviation from at least two independent experiments. The number of eggs analyzed is noted above each bar. **f** Volcano plot of gene expression microarray results. The *x*-axis shows fold change between NULL (*chk2*^*P6*^*, ote*^*PK*^*/ ote*^*B279G*^ and *ote*^*PK*^*/ chk2*^*P30*^*, ote*^*B279G*^), and HET (*chk2*^*P6*^*, ote*^*PK*^*/ CyO* and *chk2*^*P30*^*, ote*^*B279G*^*/ CyO*) ovaries and the *y*-axis shows one-way ANOVA *p* value; areas corresponding to twofold change cutoff and *p* < 0.05 are shaded, points corresponding to significantly decreasing or increasing RNAs are colored blue and red, respectively

Nuclear architecture defects are common in premature aging syndromes such as progeria^27^. Accumulating evidence suggests that compromised nuclear architecture coincides with altered DNA replication and damage repair, two processes regulated by lamins^28,29^. Indeed, studies of progeroid cells with defective maturation of prelamin A found evidence of DNA damage and activation of DDR-mediated cellular senescence^20,22^. Based on shared progeroid-like nuclear phenotypes, we tested whether *ote*^*−/−*^ GSC death might result from DDR activation, by genetically compromising the DDR pathway. The DNA damage response in GSCs depends upon activation of the transducer kinase Chk2^30,31^. For this reason, we tested the role of Chk2 on *ote* mutant phenotypes by generating double mutant animals carrying null mutations in both *ote* and *chk2* (also known as *mnk, loki* in Drosophila; Supplementary Table 1). Ovaries from wild-type and mutant animals were stained with antibodies against Vasa to identify germ cells and DAPI to visualize DNA (Fig. 2a). As previously observed^12,15^, *ote* mutant ovaries were rudimentary and lacked the strings of developing egg chambers found in wild-type ovaries. Strikingly, loss of one or both copies of *chk2* in an *ote* mutant background restored oogenesis, producing large ovaries filled with developing egg chambers (Fig. 2a). As Chk2 transduces the checkpoint through phosphorylation of downstream targets^32^, we tested whether the Chk2 kinase activity was required for execution of *ote* mutant phenotypes. To this end, we crossed a *chk2* kinase dead [*chk2*^*KD*^] allele into an *ote* mutant background. We found that both heterozygous and homozygous *chk2*^*KD*^ mutations rescued oogenesis (Fig. 2b). These data demonstrate that the kinase activity of Chk2 is crucial for *ote* mutant GSC loss.

We next completed several experiments to define the scope of the dramatic rescue in *chk2, ote* mutants. First, egg production was quantified. These analyses revealed that fecundity remained strong throughout the entire 8-day assay period (Fig. 2c). These studies  also revealed that the Chk2 rescue was dose dependent, wherein loss of one wild-type *chk2* allele partially rescued fecundity and loss of both wild-type alleles provided full rescue. Second, the composition of germaria was determined. In these experiments, ovaries were stained with antibodies against Vasa and the cytoskeletal protein Spectrin (Supplementary Fig. 1A). Spectrin distinguishes the differentiation status of germ cells. In GSCs and their immediate daughters, Spectrin is found within a spherical structure called the spectrosome. Upon differentiation, Spectrin adopts a branched shape within a structure called the fusome^33^. Germaria from newly eclosed *ote*^*+/+*^ females carry approximately four germ cells with spectrosomes, corresponding to GSCs and daughter cystoblasts, as well as multiple developing cysts with fusomes (Supplementary Fig.1A, B). In contrast, newly eclosed *ote*^*−/−*^ ovaries show a complex germarial phenotype^12^, including germaria without germ cells due to GSC death (~24%) and germaria containing only spectrosome-positive germ cells (~68%) due to blocked differentiation (Fig. 2d, Supplementary Fig. 1A). Loss of Chk2 alone has no effect on GSC homeostasis (Fig. 2d, Supplementary Fig. 1B), consistent with previous findings^31^. In *chk2, ote* double mutant ovaries, all germaria had GSCs, cystoblasts, and differentiating germ cells (Fig. 2d). Remarkably, even loss of one wild-type *chk2* allele fully rescued GSC death and restored germline differentiation within the majority (~80%) of germaria (Fig. 2d). In *chk2*^*−/−*^*, ote*^*−/−*^ germaria, there is a slight increase in the number of spectrosome-containing germ cells per germarium (Supplementary Fig. 1B), suggesting that germ cell differentiation might be delayed. Together, these data reveal loss of Chk2 largely rescues germarial phenotypes in the *ote* mutants.

### Rescued oogenesis shows minimal changes in transcription

The *chk2*-dependent rescue provided the first opportunity to assess the Ote requirement in later stages of oogenesis. This multi-faceted developmental program includes rapid syncytial germ cell divisions, oocyte specification, meiosis, endocyclic DNA replication in nurse cells, axis specification, and active transcription to produce maternal products required for early embryogenesis. We stained *chk2*^*−/−*^*, ote*^*−/−*^ ovaries with Vasa and DAPI to examine oocyte development. Overall, oogenesis appeared normal in *chk2, ote* mutant females, with only a small number (10%) of developing *chk2*^*−/−*^*, ote*^*−/−*^ egg chambers containing a reduced number of nurse cells compared to wild-type controls. Next, we examined eggs laid by *chk2, ote* mutant females. Although these eggs were phenotypically normal, all failed to hatch even when the oocyte was fertilized with an *ote*^*+/+*^ sperm (Fig. 2e). These findings imply that Ote is either required for early events in embryogenesis or that underlying defects remain in *ote, chk2* germ cells that prevent embryogenesis. To determine whether blocked embryonic development was caused by altered production of maternal RNAs, we defined effects of Ote loss on transcription. To control for inter-strain variability and ovary size, total RNA was isolated from ovaries of two *chk2*^*+/–*^*, ote*^*−/−*^ (null) backgrounds and their *chk2*^*+/–*^*, ote*^*+/–*^ (het) siblings. Surprisingly, Ote loss has minimal effects on transcription of protein-coding genes. We identified only 28 differentially expressed genes (5 downregulated and 23 upregulated) in *ote* null ovaries (Fig. 2f, Supplementary Fig. 1C). Gene ontology analyses revealed a modest enrichment for serine endopeptidases among the upregulated genes and unsupervised hierarchical clustering revealed no distinct *ote* mutant signature (Supplementary Fig. 1C, D). These data indicate that the maternal RNA pool is largely unaffected in *ote* mutants, with the exception of *ote* mRNA. Notably, early embryos possess a large pool of maternally deposited Otefin that was linked to nuclear envelope assembly^34^, suggesting that embryogenesis might fail due to the absence of Ote itself. Even so, oogenesis in *chk2, ote* mutant females proceeds with remarkably few detectable defects.

### Loss of ATR rescues oogenesis in *ote* mutants

**Fig. 3:**
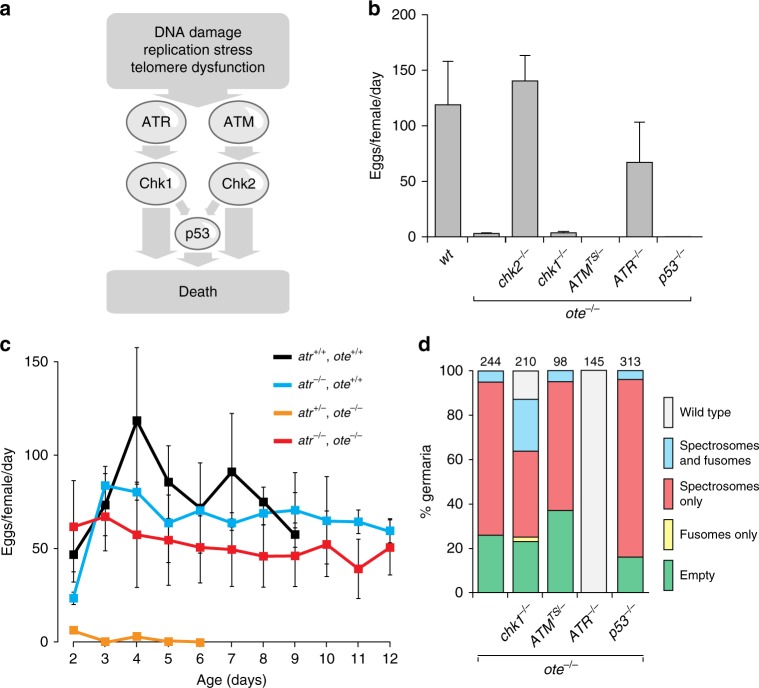
Select DDR components contribute to the checkpoint pathway in *ote* mutants. **a** Schematic representation of signaling through the DNA damage response (DDR) pathway. Common triggers that activate the ATM and ATR responder kinases are shown. The responder kinases canonically activate the Chk2 and Chk1 transducer kinases, which signal to p53 and cause cell death. **b** Quantification of peak fecundity (eggs per female per day) of *ddr, ote* double-mutant females crossed to *ote*^*+/+*^ males. Genotypes are noted below each bar, with *ote*^*−/−*^ corresponding to *ote*^*B279G/PK*^. Error bars represent the standard deviation from a minimum of two independent experiments, each using 7–15 females. **c** Fecundity (eggs per female per day) of *ddr, ote* double-mutant females of indicated genotypes, crossed to wild-type males, with *ote*^*−/−*^ corresponding to *ote*^*B279G/PK*^. Genotypes are noted above the graph. Error bars indicate the standard deviation from a minimum of three independent experiments with 7–15 females each. Wild-type data from Fig. 2c. were included in the graph as a reference. **d**. Quantification of germarial phenotypic classes (noted to the right) in 2-day-old *ddr, ote* double-mutant females, with *ote*^*−/−*^ corresponding to *ote*^*B279G/PK*^. Number of germaria assessed from at least ten females is noted above each bar

To understand the extent of the DDR pathway involvement in *ote* mutants, we generated double mutants lacking Ote and DDR components, and examined effects on female fecundity and germarial phenotypes. We tested mutations in genes encoding both responder kinases, *ATM* (*tefu*), and *ATR* (*mei-41*), the second transducer kinase *Checkpoint kinase 1* (*Chk1*; *grapes*), and the downstream effector, *p53* (Fig. 3a, Supplementary Table 1). We reasoned that if a DDR component contributed to signaling in the GSC checkpoint, then germ cell phenotypes would improve in the *ote, ddr* double mutants relative to *ote* single mutants. First, we examined effects of loss of ATM. As complete loss of ATM is lethal in Drosophila, temperature sensitive ATM alleles were used. We found no rescue of egg laying or germarial phenotypes in ATM-defective *ote* mutants (Fig. 3b–d), suggesting that ATM is not a component of the GSC checkpoint. Second, we tested effects of loss of the second responder kinase, ATR. Interestingly, oogenesis was rescued in the *atr*^*−/−*^*; ote*^*−/−*^ females, with egg laying sustained throughout the 12-day measurement period (Fig. 3b, c). Germarial phenotypes in *atr*^*−/−*^*; ote*^*−/−*^ ovaries were completely rescued, with all germaria carrying GSCs, cystoblasts and differentiating germ cells (Fig. 3d). These data indicate that ATR is the responder kinase of the checkpoint resulting from Ote loss. Third, we evaluated effects of loss of the second transducer kinase, Chk1. Whereas no rescue of fecundity was found in *chk1*^*−/−*^*, ote*^*−/−*^ females (Fig. 3b), a modest improvement was observed in germline differentiation (Fig. 3d). These data suggest that Chk1 has a minor role. Finally, we tested effects of loss of the DDR effector p53. We found no rescue of fecundity or germarial phenotypes, including no improvement of GSC survival (Fig. 3b–d). These data are consistent with the absence of staining with standard apoptosis markers in *ote*^*−/−*^ germaria^12,15^, and support previous studies showing that GSC death is distinct from classic apoptotic cell death^35^. Together, our data suggest that a checkpoint is activated in GSCs in the absence of Ote, wherein ATR is the critical responder kinase and Chk2 is the critical transducer kinase.

### The GSC checkpoint is activated by a non-canonical trigger

**Fig. 4:**
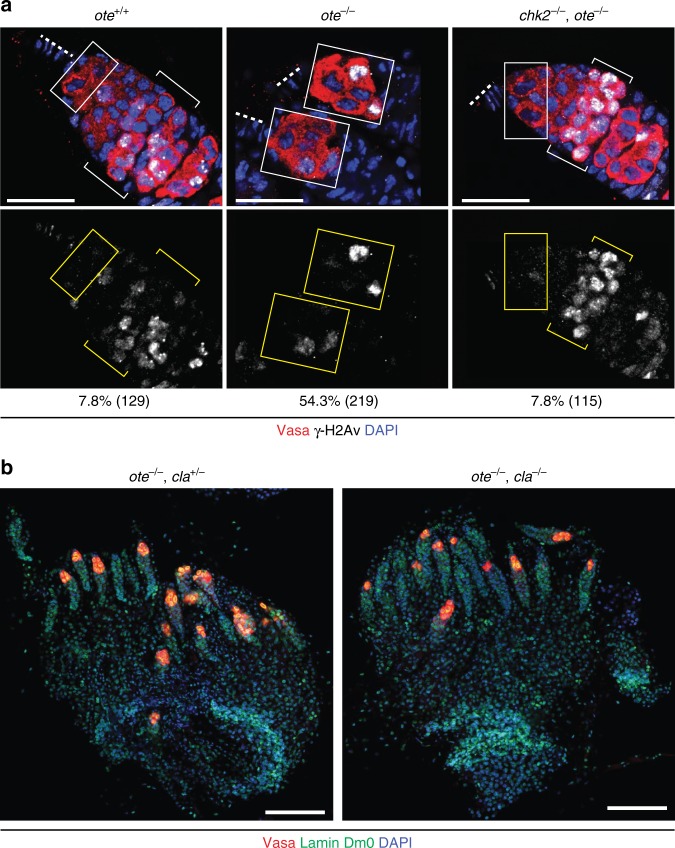
DNA damage in *ote*^*−/−*^ GSCs is downstream of Chk2. **a** Confocal images of germaria stained with antibodies against Vasa (red), DNA damage marker γ-H2Av (grayscale) and with DAPI (blue). Genotypes are noted above each image, wherein *ote*^*−/−*^ corresponds to *ote*^*B279G/PK*^ and *chk2*^*−/−*^ corresponding to *chk2*^*P6/P30*^. Bottom: Image of the signal in the γ-H2Av channel. Boxes and brackets indicate GSCs and meiotic germ cells, respectively. Dashed line indicates the position of GSC niche. The percentage of γ-H2Av-positive GSCs is noted at the bottom of the γ-H2Av image, with the number of GSCs analyzed in parenthesis. All scale bars represent 20 µm. **b** Confocal images of whole mount ovaries dissected from *ote* mutants with heterozygous or homozygous loss of Claspin (Cla). Ovaries were stained for Vasa (red), Lamin Dm0 (green), and DNA (DAPI, blue), revealing the absence of suppression of *ote* mutant phenotypes. Genotypes are indicated at the top of each image, with *ote*^*−/−*^ corresponding to *ote*^*B279G/PK*^; scale bars represent 100 µm

DNA double-strand breaks activate the DDR pathway in GSCs^30^. To test the role of DNA damage in Chk2-dependent GSC loss, we stained *ote*^*+/+*^ and *ote*^*−/−*^ ovaries with antibodies against γ-H2Av (the homolog of mammalian γ-H2AX), a commonly used marker of DNA damage^30,36,37^. In wild type germaria, γ-H2Av accumulates in the nuclei of all meiotic germ cells but is infrequently detected in GSCs^30,31^. However, upon DNA damage, GSCs rapidly respond, such that nearly all (~95%) stain with γ-H2Av antibodies within 1 hour of induction of double-strand breaks (DSBs), persisting for several days^30^. Consistent with these studies, we found that γ-H2Av accumulated in all meiotic germ cells and confirmed a low frequency in wild-type GSCs (7.8%; Fig. 4a). In *ote*^*−/−*^ GSCs, over half (54.3%) had γ-H2Av signal, indicating that these GSCs have increased levels of DNA damage.

We conducted genetic epistasis studies to test the role of DNA damage in checkpoint activation. Phosphorylation of H2AX occurs upstream of Chk2 activity and depends upon the ATM/ATR kinases^38^. Thus, we reasoned that if DNA damage triggered the DDR pathway, then *chk2*^*−/−*^*, ote*^*−/−*^ GSCs would maintain elevated frequencies of γ-H2Av accumulation similar to those found in *ote*^*−/−*^ GSCs. Contrary to this prediction, we found that the frequency of γ-H2Av staining in *chk2*^*−/−*^*, ote*^*−/−*^ GSCs reverted to wild-type (7.8%), even though all *chk2*^*−/−*^*, ote*^*−/−*^ germaria were γ-H2Av-positive in the meiotic zone (Fig. 4a). We note that this response differs from one elicited by DNA damage, wherein *chk2*^*−/−*^ GSCs retain elevated frequencies of γ-H2Av signal^31^. These surprising results were reproduced using a second, independently generated γ-H2Av antibody (Supplementary Fig. 2A). Our data indicate that increased levels of γ-H2Av in *ote*^*−/−*^ GSCs are downstream of Chk2, suggesting that DNA damage is not the checkpoint trigger.

In complementary analyses, we investigated whether loss of Ote compromised DNA repair. Unrepaired meiotic DSBs cause retention of γ-H2Av staining within the oocyte nucleus of developing egg chambers [*spnB* mutants in Supplementary Fig. 2B)^39^. We examined γ-H2Av staining in *chk2*^*−/−*^*, ote*^*−/−*^ oocyte nuclei of similarly aged egg chambers, finding that the γ-H2Av signal was lost (Supplementary Fig. 2B). These studies show that loss of Ote does not affect meiotic DSB repair.

Replication stress activates ATR and Chk2 in the Drosophila germline^40^. Notably, mutations in lamin disrupt the distribution of replication factories and induce replication fork arrest, causing replication stress^28,41^. Motivated from the deformed NL in *ote*^*−/−*^ GSCs (Fig. 1b), we tested whether loss of Ote induces replication stress. To this end, we assessed the role of Claspin, a conserved component of DNA replication stress pathways^42,43^. In Drosophila, Claspin-mediated signaling occurs in response to incomplete DNA replication, but not DNA DSBs^43^. Furthermore, replication stress in the ovary produces atrophied ovaries, defects that are suppressed by loss of Claspin^40^. Using previously tested alleles^40^ (Supplementary Table 1), we generated *ote*^*−/−*^*; claspin*^*–/+*^ and *ote*^*−/−*^*; claspin*^*−/−*^ females and assessed ovarian phenotypes. Unlike the dramatic rescue observed with ATR and Chk2 loss, the *ote, claspin* double-mutant ovaries remained rudimentary, with nearly 40% of germaria devoid of germ cells (Fig. 4b). Whereas GSC loss resulting from replication stress is partially dependent on p53^40^, GSC loss in *ote*^*−/−*^ mutants is p53-independent (Fig. 3b, d). Based on these data, we conclude that replication stress is not responsible for induction of ATR/Chk2-dependent GSC loss in *ote* mutants.

**Fig. 5:**
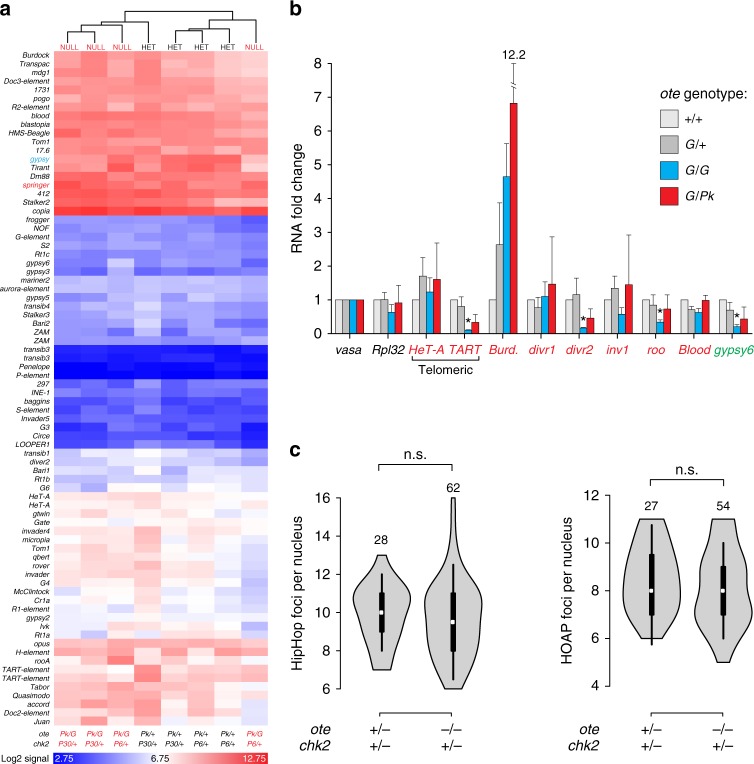
Transposon transcription and telomere capping are unchanged in *ote* mutants. **a** Heat map of the log2 signal intensities of RNAs corresponding to *D. melanogaster* transposons (indicated to the left); unsupervised clustering reveals no segregation by genotype. The genotypes are shown below. **b** Quantitative RT-PCR analysis of transposon RNAs in the ovary. For Ote-containing genotypes (light and dark gray), ovaries were dissected from less than two-hour old females, and for *ote* null genotypes (blue and red), ovaries were dissected from 3-day-old females. Transposons analyzed are noted below (red indicates germline transposons and green indicates somatic transposons). RNAs were normalized to *vasa* and fold change is shown relative to *ote*^*+/+*^. Error bars indicate standard deviation from three biological replicates (*p < 0.05, Student’s *t*-test comparing RNAs from *ote* mutant ovaries to RNAs from *ote*^*B279G/+*^ ovaries). **c** Violin plots of the number of HipHop foci (left) or HOAP foci (right) per GSC nucleus in 1-day-old females. Numbers of GSCs analyzed are noted above each plot. Significance was assessed by Student’s *t*-test; n.s., not significant

We next explored other possible mechanisms known to activate ATR/Chk2. Germline activation of these kinases has been linked to transposon de-repression and mobilization^44–46^. The presence of transposon-mapped probes within our microarrays provided an opportunity to investigate effects of Ote loss on transcription of transposons. Of the 73 transposons annotated on the array, only two demonstrated modest differences in *chk2, ote* double-mutant ovaries, corresponding to *gypsy* (3.4-fold downregulation) and *springer* (2.1-fold upregulation). As with the protein-coding genes, unsupervised hierarchical clustering did not reveal an *ote* mutant signature in transposon expression (Fig. 5a), implying that transposons remain stable and do not activate the checkpoint in *ote*^*−/−*^ ovaries. To complement these analyses, we directly examined the expression and function of several piRNA-regulated transposons (^47,48^; Fig. 5b). These included the specialized retrotransposons that maintain Drosophila telomeres (*HeT-A* and *TART*) because telomere damage activates Chk2^49^ and increased transcription of telomeric transposons is a hallmark of telomere dysfunction^50^. We completed quantitative reverse transcriptase PCR (RT-PCR), using two wild-type and two *ote* mutant backgrounds, with animals of three of these genetic backgrounds generated from a cross with the same *ote*^*B279G*^ stock. In this way, differences in transposon position and numbers were minimized. We reasoned that if loss of Ote caused transposon mis-regulation, then both *ote* mutant backgrounds should show consistent changes in gene expression. Our studies revealed largely unchanged levels of retrotransposon and telomeric RNAs in *ote* mutants relative to controls, with no transposon showing significant changes in both mutant backgrounds (Fig. 5b). Further, we examined telomere organization directly. Drosophila telomeres are capped with the Terminin complex, composed of HP1a, HipHop, and HOAP^51^. GSCs have 7–14 Terminin foci, depending on the stage of the cell cycle^24,52^. Using HipHop and HOAP antibodies, we observed no significant difference between *ote*^*–/+*^ and *ote*^*−/−*^ profiles (Fig. 5c). Taken together, these data argue that *ote* mutants retain transposon stability and telomere function, implying these factors do not induce Chk2 activity in *ote* mutant GSCs.

### NL defects persist in *chk2, ote* mutant GSCs

**Fig. 6:**
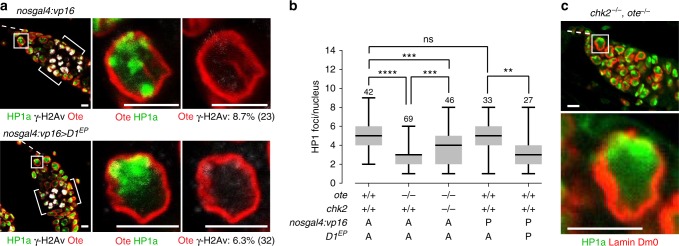
HP1a coalescence does not lead to increased γH2Av staining or GSC loss. **a** Confocal images of germaria and GSC nuclei in females carrying either the *nosgal4:vp16* driver alone (top) or the *nosgal4:vp16* and the *D1*^*EP473*^ allele (bottom). Ovaries were stained with antibodies against HP1a (green), γ-H2Av (white), and Ote (red). The percentage of γ-H2Av-positive GSCs is noted at the bottom of the GSC image, with the number of GSCs analyzed in parenthesis. Scale bars represent 5 μm. **b** Box plots of the quantification of HP1a foci per GSC nucleus found in GSCs of the indicated genotype. Genotypes are noted below each box plot, and the number of nuclei analyzed is noted above each top whisker. An A indicates absence of the *nosgal4:vp16* driver or the *D1*^*EP*^ allele, and P indicates their presence. For each box plot, the box represents the 25th to 75th percentile interval, the line represents the median and the whiskers represent the 5th to 95th percentile interval and non-outlier range. Asterisks indicate significance (one-way ANOVA, ns = not significant, **<0.01, ***<0.001, ****<0.0001). **c** Confocal images of a GSC nucleus from 1-day-old *chk2*^*−/−*^*, ote*^*−/−*^ females stained for Lamin Dm0 (red) and HP1a (green), revealing that nuclear structural defects are upstream of Chk2. Genotypes are listed above each image. The *chk2*^*−/−*^ corresponds to *chk2*^*P6*^*/chk2*^*P30*^ and *ote*^*−/−*^ corresponds to *ote*^*B279G/PK*^. The box outlines GSCs. Scale bars represent 5 μm

Altered chromatin and chromosome structures can promote DNA damage-independent DDR signaling^53–55^. These observations prompted our investigation of whether heterochromatin coalescence represents the trigger of Chk2-dependent GSC loss in *ote* mutants. Although tools for overexpression of HP1a in the male germline exist^56^, these did not establish overexpression of HP1a in the female germline. Instead, we used a complementary approach to promote heterochromatin aggregation. Overexpression of the multi-AT hook satellite DNA binding protein D1 induces ectopic chromosome associations^57^, suggesting that elevated levels of D1 in GSCs might promote centromeric heterochromatin interactions and coalescence. To this end, we overexpressed D1 using a *nos-gal4:VP16* driver to direct expression of the *D1*^*EP473*^ responder allele (Supplementary Fig. 3A), an allele that carries a GAL4-dependent *P{EP}* element in the 5′ UTR of the *D1* gene^57^. Strikingly, *nos-gal4:VP16*, *D1*^*EP473*^ GSCs had elevated D1 levels and  HP1a aggregation, similar to levels found in *ote*^*-/-*^ GSCs (Fig. 6a, b, Supplementary  Fig. 3A). Even so, the NL structure was unaffected, as shown by Ote staining (Fig. 6a). Despite increased HP1a coalescence, GSCs survive, γH2Av levels are low (6.3%) and oogenesis is normal (Fig. 6a, Supplementary Fig. 3B). These observations indicate that HP1a coalescence alone does not cause Chk2-dependent germ cell death. Although D1 and Ote loss might affect HP1a chromatin structure in different ways, mechanistically distinct perturbations of chromatin structure elicit DDR responses^53–55^ suggesting that, in GSCs, chromatin-based mechanisms of DDR signaling are not responsible for checkpoint activation.

We investigated whether nuclear defects remain in *ote*^*−/−*^*, chk2*^*−/−*^ ovaries (Fig. 6c). Quantification of HP1a foci revealed an intermediate phenotype in *ote*^*−/−*^*, chk2*^*−/−*^ GSCs (Fig. 6b). These data suggest that Chk2 is upstream of HP1a coalescence and might contribute to heterochromatin clustering. In contrast, NL deformities remain in *ote*^*−/−*^*, chk2*^*−/−*^ GSCs (Fig. 6c), implying that these defects are upstream of Chk2. Taken together, our collective epistasis studies suggest that alteration of NL structure is the primary event that leads to checkpoint activation in *ote* mutants.

### Chk2 rescue reveals cell-type specificity of NL checkpoint

**Fig. 7:**
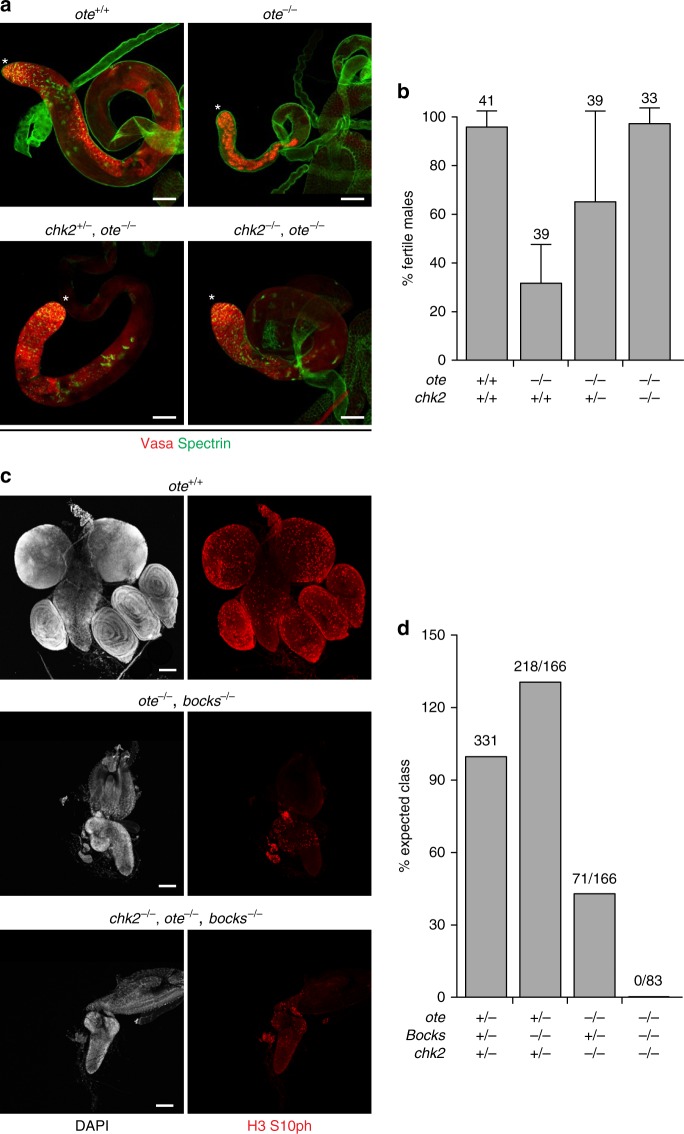
Checkpoint inhibition rescues male GSC, but not somatic defects in *ote* mutants. **a** Confocal images of testes from 6-day-old males stained for Vasa (red) and Spectrin (green). Genotypes are noted above the images. The GSC niche is denoted by an asterisk. **b** Percentage of fertile 12–15-day-old males mated to *ote*^*+/+*^ virgin females. Males were determined to be fertile if greater than 5 offspring were produced over a 3-day mating period as described in ref. ^16^. Genotypes are noted below, and the number of males analyzed is noted above each data set. Error bars indicate the standard deviation from a minimum of three independent experiments. **c** Confocal images of the central nervous system and surrounding imaginal discs dissected from wandering third instar larvae stained for DAPI (gray) and antibodies against phosphorylated serine 10 of Histone H3 (H3 S10ph; red). Genotypes are noted above each image. All scale bars are 100 µm. **d** Percent expected class from the following cross: *y*^*1*^*,w*^*67c23*^*; chk2*^*P6*^*,ote*^*PK*^*/CyO; bocks*^*Δ66*^*/Tm6b,Tb*^*1*^ crossed to *y*^*1*^*,w*^*67c23*^*; chk2*^*P30*^*,ote*^*B279G*^*/CyO; bocks*^*Δ10*^*/Tm6b,Tb*^*1*^. The genotype of the progeny is noted below and the number of progeny eclosed over the expected number is noted above

Requirements for Ote extend beyond the female germline. Males lacking Ote also display age-dependent GSC loss and sterility^16^. In the testis, spermatogenesis initiates within a single stem cell niche, called the hub, that supports two stem cell populations, germline stem cells (GSCs) and cyst stem cells (CySCs). Both stem cell populations undergo asymmetric division to produce differentiating daughters that move away from the hub, forming a unit of two post-mitotic somatic cyst cells and one germ cell. Subsequent mitotic and meiotic germ cell divisions produce a unit comprised of 64 spermatids that proceed to differentiate into sperm. To determine whether male germline defects are caused by checkpoint activation, two assays were completed. First, testes phenotypes were defined. As reported previously^16^, 6-day-old wild-type testes had Vasa-positive germ cells at all stages of spermatogenesis, whereas 6-day-old *ote*^*−/−*^ testes showed GSC loss and blocked differentiation (Fig. 7a). In the aged *chk2*^*−/−*^*, ote*^*−/−*^ testes, we found that GSCs were retained and germ cells differentiated (Fig. 7a), indicating rescued gametogenesis. Second, male fertility was assessed using a sperm depletion assay^16^. Whereas only ~30% of 2-week-old *ote*^*−/−*^ males remained fertile, 2-week-old *chk2, ote* double-mutant males showed dose-dependent improvement of fertility, with *chk2*^*−/−*^*, ote*^*−/−*^ males reaching wild-type levels (Fig. 7b). These data reveal a Chk2-dependent GSC loss and sterility in *ote*^*−/−*^ males. Strikingly, *ote*^*−/−*^ male GSCs have an irregular and thickened NL (Supplementary Fig. 4), consistent with NL dysfunction as a primary event in checkpoint activation.

Ote is required for the development of somatic tissues. This requirement was uncovered in genetic studies testing overlapping functions of NL LEM-D proteins^13^. Indeed, *ote*^*−/−*^*; bocks*^*−/−*^ double mutants die during larval development, carrying rudimentary organs and imaginal discs, with few mitotic cells (Fig. 7c). To determine whether lethality in the *ote, bocks* double mutants is Chk2-dependent, we assessed effects of its loss. To this end, we generated *chk2*^*−/−*^*, ote*^*−/−*^*; bocks*^*−/−*^ triple mutant animals. Based on the number of heterozygous sibling offspring (331) recovered from a cross of trans-heterozygous parents, 83 triple mutant adult offspring were expected if lethality were fully rescued. In contrast to this prediction, no *chk2*^*−/−*^*, ote*^*−/−*^*; bocks*^*−/−*^ adults were found (Fig. 7d). In addition, *chk2*^*−/−*^*, ote*^*−/−*^*; bocks*^*−/−*^ triple mutant larvae carried rudimentary brains and imaginal discs with reduced levels of mitotic nuclei, revealed with antibodies against phosphorylated serine 10 of Histone H3 (H3 S10ph; Fig. 7c). Indeed, *chk2*^*−/−*^*, ote*^*−/−*^*; bocks*^*−/−*^ triple mutant phenotypes were indistinguishable from *ote*^*−/−*^*; bocks*^*−/−*^ double mutants. These data show that Chk2 loss is not sufficient to rescue the somatic requirements of Ote and Bocks, demonstrating GSC-specific rescue.

## Discussion

**Fig. 8:**
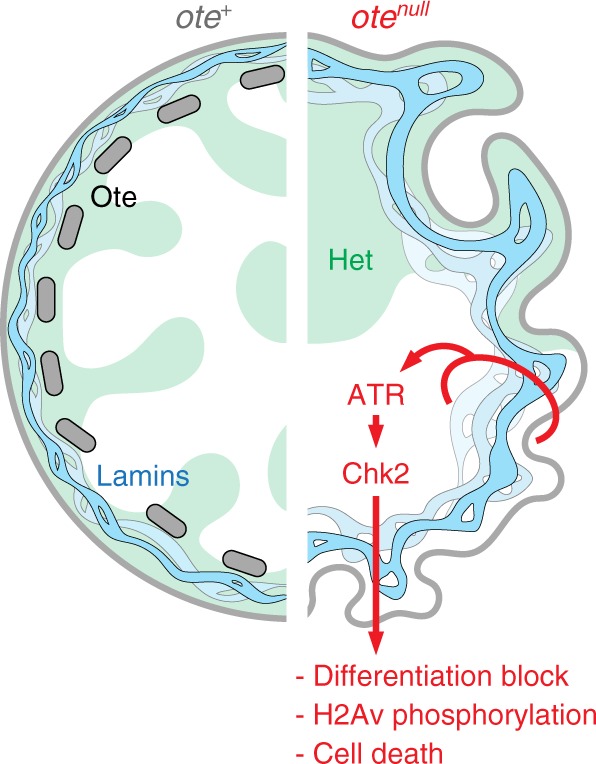
Loss of Otefin activates a NL checkpoint in GSCs. In *ote*^*+/+*^ GSCs (left side), Ote (gray bars) resides within the NL, stabilizing NL structure (blue ribbon) and heterochromatin (green shading) contacts. In *ote*^*−/−*^ GSCs (right side), the NL becomes thick and deformed, causing NL dysfunction that activates a checkpoint pathway. This pathway uses the ATR and Chk2 kinases, leading to blocked differentiation, H2Av phosphorylation, and, ultimately, death of GSCs. As loss of ATR/Chk2 in *ote* mutants rescues oogenesis, but not embryogenesis, we suggest this NL checkpoint pathway functions to ensure that healthy gametes are passed on to the next generation

The Drosophila emerin homolog Ote has an essential requirement for GSC survival and germ cell differentiation^12,16^. Here, we show that Ote loss causes GSC-specific nuclear defects that include a thickened and irregular NL and aggregation of heterochromatin (Fig. 1). Strikingly, inactivation of two DDR kinases, ATR, and Chk2, rescues oogenesis in *ote*^*−/−*^ females (Figs. 2, 3), a rescue that is cell-type specific (Fig. 7). Genetic and cytological features of the checkpoint pathway present in *ote* mutant GSCs differ from those found in canonical DDR pathways (Figs. 3, 4, 5;^58^). In addition, although heterochromatin coalesce is present, such defects by themselves do not trigger the checkpoint (Fig. 6, S3). Instead, our data correlate Chk2 activity with defects in NL structure (Fig.1, S4), indicating that NL dysfunction is responsible for the activation of a checkpoint pathway in GSCs (Fig. 8). Despite remarkably normal oogenesis, rescued oocytes do not support embryogenesis (Fig. 2e). We suggest this NL checkpoint pathway functions in GSCs to ensure that only healthy gametes are passed on to the next generation.

Our studies identify ATR as the critical responder kinase and Chk2 as the critical transducer kinase in the NL checkpoint (Figs. 2, 3, and 6). This signaling axis differs from the canonical ATM-to-Chk2 or ATR-to-Chk1 axes^58^. Several factors might contribute to the choice of responder and transducer kinase. First, species-specific constraints might exist. ATR, but not ATM is essential in mammals, whereas ATM, but not ATR, is essential in Drosophila^59,60^. Second, cell-type specific distinctions are apparent. In both the fly and mouse germline, persistent meiotic double-strand breaks activate ATR and Chk2^45,61^, implying that the ATR–Chk2 axis might be dominant in germ cells. Third, the nature of the trigger might influence which proteins are involved in signaling. For example in Drosophila, ATR and Chk2 are both required for the patterning defects caused by a failure to repair meiotic double-strand breaks^62^. However, in DNA-damaged GSCs, ATR protects against GSC death, whereas Chk2 promotes it^30^. Our data suggest that in the case of the NL checkpoint, both ATR and Chk2 promote germ cell death. These studies add to growing evidence that the DDR pathway is modular, with selective use of pathway components in response to various cellular stresses^58^.

Activated Chk2 is commonly associated with phosphorylation and activation of p53^63^. Canonically, p53 activation leads to cell cycle arrest and apoptosis^64^. Our studies demonstrate that GSC loss persists in *ote*^*−/−*^*; p53*^*−/−*^ females (Fig. 3b, d), suggesting that classical apoptosis is not responsible for GSC death. These findings are consistent with the absence of classic markers of apoptosis in *ote* mutants^15^ and observations that the p53 regulatory network differs in GSCs^65^. Recently, an alternative cell death pathway was identified in spermatogonia of Drosophila testes^66^. This pathway is responsible for spontaneous elimination of spermatogonia, using activated lysosomal and mitochondrial-associated factors. Additional studies are needed to determine whether ATR/Chk2-dependent GSC loss in *ote* mutants targets a similar pathway.

Our data suggest that NL dysfunction is the primary cause of the ATR/Chk2 checkpoint in GSCs. Notably, NL defects are found only in affected cells and persist in rescued *chk2*^*−/−*^*, ote*^*−/−*^ double mutants (Figs. 1 and 6; Supplementary Fig. 4). Multiple mechanisms might connect nuclear architecture changes to ATR/Chk2 activation. First, altered NL structure might change genomic contacts needed for appropriate transcriptional regulation, with resulting gene expression changes prompting activation of the checkpoint. While we did not observe global transcriptional changes during oogenesis (Fig. 2f), identification of transcriptional changes specific to GSCs or early germ cells would have been masked in our studies. Second, disruptions in the NL might affect trafficking of products between the nucleus and cytoplasm. Notably, a recent study identified large ribonucleoparticles (megaRNPs) that exit the nucleus by egress or budding through the inner and outer nuclear membranes^67^, a process disrupted by defects in the NL^68^. As such, it remains possible that the thickened NL in *ote*^*−/−*^ GSCs disrupts megaRNP egress, leading to cellular stress and ATR/Chk2 activation. Third, defects in the NL structure might alter scaffolding of components of the DDR pathway, leading to checkpoint activation. Indeed, proteomic studies from Drosophila cultured somatic cells found that Ote interacted with proteins involved in DNA replication and repair^69^, implying that Ote might assemble responder and transducer kinases complexes at the NL. However, observations that the ATR/Chk2-dependent checkpoint is GSC-specific (Fig. 7c, d), coupled with findings that meiotic double-strand breaks are repaired appropriately in *chk2*^*−/−*^*, ote*^*−/−*^ germaria (Supplementary Fig. 2B), argue against this model. Fourth, structural alteration in the nuclear envelope itself might trigger ATR/Chk2 activation^70^. Indeed, emerging evidence implicates ATR as a general sensor of the structural integrity of cellular components^71^. Further studies are needed to identify how NL dysfunction triggers the GSC-specific checkpoint.

Mutations in NL LEM-D proteins cause dystrophic diseases. Much evidence suggests that these diseases result from compromised stem cell populations that underlie the defects in tissue homeostasis^72–75^. Indeed, a wealth of evidence links NL defects to increased DNA damage^20,22,41,76,77^. Our data are consistent with these reports, as we show that elevated accumulation of the commonly used DNA damage marker. However, we find that phosphorylation of the H2A variant occurs downstream of Chk2, suggesting that accumulation of DNA damage in cells with a dysfunctional NL might be a consequence of cells dying, not the primary cause. These unexpected results suggest that caution is needed in linking causation of γH2Av/H2X accumulation to DNA damage and a failure in DNA repair. Indeed, recent studies of progerin-expressing cells indicated that the cellular defect in Hutchinson–Gilford progeria cells does not lie in defective DNA repair and DNA damage, even though these cells accumulate phosphorylated H2AX^78^. Our findings establish a new context for consideration of mechanisms of laminopathic diseases, suggesting that detrimental effects of NL dysfunction are primary events that are linked to checkpoint activation and stem cell loss.

## Methods

### Animal culture conditions

Drosophila crosses were incubated at 25 °C at 70% humidity on standard cornmeal/agar medium with p-hydroxybenzoic acid methyl ester as a mold inhibitor. For fecundity assays, animals were supplied dry active yeast pellets. The wild-type reference strain was *y*^*1*^*, w*^*67c23*^. In all cases, *ote* mutant animals were *y*^*1*^*, w*^*67c23*^*; ote*^*B279G/PK*^, wherein both alleles fail to generate Ote protein^12^. The *ote*^*B279G*^ allele carries an insertion of a Piggybac transposon at +764 and *ote*^*PK*^ carries a premature stop at codon 127^12^. Other alleles used are listed in Supplementary Table 1, and genotyping primers are listed in Supplementary Table 3. In all cases, mutant animals carried heteroallelic combinations of alleles to avoid complications associated with homozygous chromosomes that carry second site mutations.

### Immunohistochemical analyses

Adult Drosophila ovaries were dissected and stained as described in ref. ^79^. Wandering third instar larval tissues were processed similarly to adult ovaries with one additional permeabilization step with 0.1% TritonX-100 in 1X PBS for 1 h at room temperature immediately following fixation. Primary antibodies were diluted in 5% BSA, 0.3% TritonX-100 in PBS and are listed in Supplementary Table 2. Alexa Fluor-conjugated secondary antibodies were used at a 1:500 dilution. All images were collected on a Zeiss LSM 710 Confocal Microscope, cropped and rotated using ImageJ software, size matched with Adobe Photoshop and assembled with Adobe Illustrator. The number of HP1a foci was quantified manually using ImageJ. Unless otherwise noted, all images are a single slice of a confocal stack.

### Analyses of ovarian phenotypes

Germarial phenotypes were quantified in 2-day-old females as described in^12^. We analyzed four to five germaria from one ovary, at least five different pairs of ovaries for each biological replicate, and at least two to three biological replicates. For female fecundity assays, 7 to 15 1-day-old females and 4 to 8 males were placed in egg collection bottles with orange juice and agar egg collection plates (90% orange juice, 0.9% agar, 1% ethyl acetate) with wet yeast. Egg collection plates were changed every 24 h. Fecundity is reported as eggs laid per female per day. If a female died during the assay, it was assumed to die minutes before changing of the egg collection plate such that the number of females in the bottle changed in the subsequent 24-h collection period. Egg collection plates were incubated at 25 °C for an additional 30 to 40 h to quantify the number of hatched progeny.

### RNA analyses

For qRT-PCR analyses, biological replicates of 25 ovary pairs were dissected in PBS on ice and stored at −80 °C. To normalize for the difference in ovary size, ovaries were dissected from <2-h-old Ote-containing and 3-day-old *ote* mutant females. RNA was isolated with TRIzol (Invitrogen) and treated with DNaseI using DNA-free kit (Ambion). cDNA was generated using the High Capacity cDNA kit with random hexamer primers (Applied Biosystems) and diluted fivefold prior to setting up qPCR reactions. Primers used in this study are listed in Supplementary Table 4. Cycle threshold levels were normalized to housekeeping gene *RpL32,* or the germline-restricted gene *vasa,* and fold enrichment was calculated using the ΔΔCt method^80^. Statistical significance was tested by comparing fold change of *ote* mutant samples to those of *ote*^*−/+*^ samples.

For microarray analyses, two biological replicates of 100 ovary pairs per genotype were dissected in PBS and stored at −80 °C. Four genotypes were studied, including two *chk2*^*+/−*^*, ote*^*−/−*^ (null) backgrounds [*chk2*^*P30*^*, ote*^*B279G*^*/ chk2*^*+*^*, ote*^*PK*^ and *chk2*^*P6*^*, ote*^*PK*^*/ chk2*^*+*^*, ote*^*B279G*^] and their *chk2*^*+/−*^*, ote*^*+/−*^ (het) siblings [*chk2*^*P30*^*, ote*^*B279G*^*/chk2*^*+*^*, ote*^*+*^ and *chk2*^*P6*^*, ote*^*PK*^*/ chk2*^*+*^*, ote*^*+*^]. Ovaries were dissected from 1-day-old females. RNA was isolated with TRIzol (Invitrogen). DNaseI treatment (Qiagen DNaseI) was done on RNeasy columns (Qiagen) during RNA purification. RNA sample quality was assessed with a Agilent 2100 Bioanalyzer prior to hybridization. Affymetrix Drosophila 2.0 arrays (900532) were used for microarray hybridization (University of Iowa Genomics Division). Array files were processed and analyzed using Affymetrix Transcriptome Analyses Console version 4.0, using default settings and RMA normalization, and contrasting four samples per group (*ote*^*null*^ vs *ote*^*het*^), using two-fold change and *p* < 0.05 (one-way ANOVA) cutoffs.

### Sperm exhaustion assay

A sperm exhaustion assay assessed male fertility, completed as described in ref. ^16^. Briefly, less than 1-day-old males were collected and individually crossed to three new newly eclosed wild-type virgin females every 3 days until the males reached an age of 15 days. A male that generated five or more progeny in 3 days was considered fertile at that age window.

### Viability analysis

To determine whether Chk2 loss rescued somatic defects associated with the absence of Ote, we assessed the viability of *chk2, ote; bocks* triple mutant animals. In these studies, we crossed *y*^*1*^*w*^*67c23*^*; chk2*^*P6*^*, ote*^*Pk*^*/CyO; bocks*^*Δ66*^*/Tm6b,Tb*^1^ males to *y*^*1*^*w*^*67c23*^*; chk2*^*P30*^*, ote*^*B279-G*^*/CyO; bocks*^*Δ10*^*/Tm6b,Tb*^1^ females. Progeny were genotyped and counted from each vial, until no progeny eclosed for 48 h.

### Statistical analysis

For microarray analyses and HP1 foci quantification, statistical significance was assessed using one-way ANOVA. For all other data, statistical significance was tested using a two-tailed Student’s *t*-test.

## Electronic supplementary material

Supplementary Information

## Data Availability

The microarray data set generated in the current study are available at Gene Expression Omnibus under the accession number GSE95309.
