# Supplementary material for: Nuclear lamina dysfunction triggers a germline stem cell checkpoint
Source: Nat Commun. 2018 Sep 27;9:3960. doi: 10.1038/s41467-018-06277-z (PMC6160405; doi:10.1038/s41467-018-06277-z)
Supplement: Supplementary file 1 — Supplementary Information [file 41467_2018_6277_MOESM1_ESM.pdf]

Nuclear lamina dysfunction

triggers a germline stem cell checkpoint

Barton *et al*.

Supplementary Figure 1

A

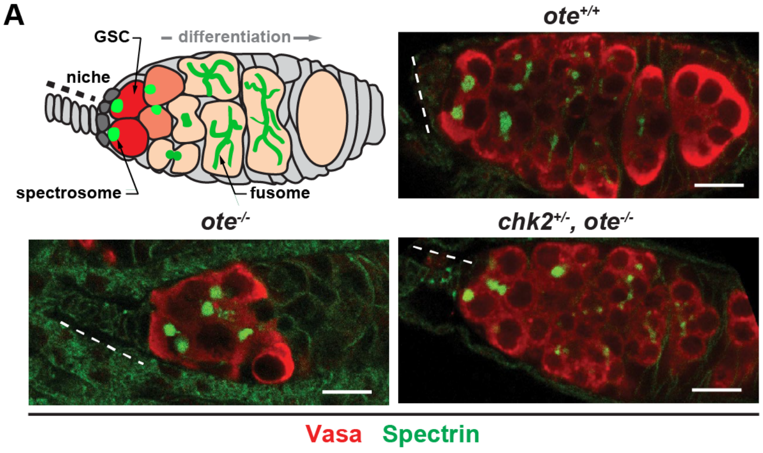

B

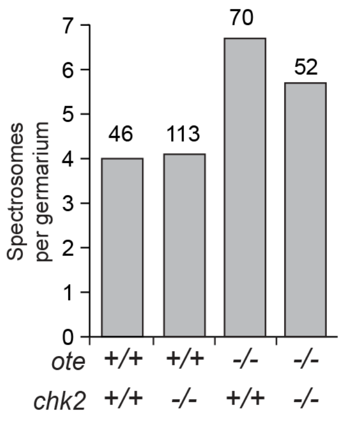

C

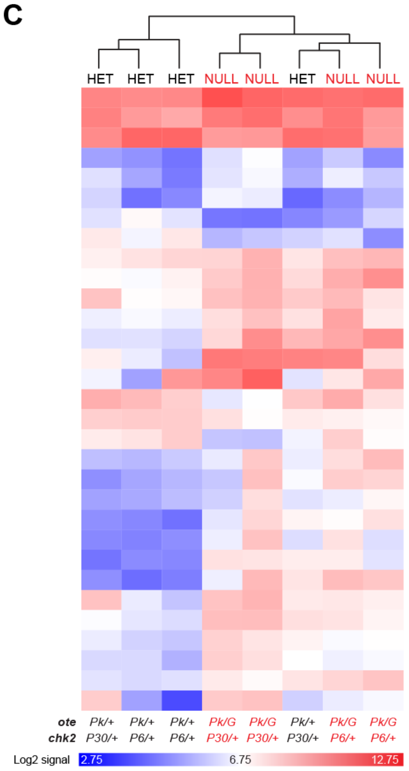

Affy ID	null avg (log2)	het avg (log2)	Fold change	ANOVA p-value	Gene symbol
1624543_s_at	11.19	10.10	2.13	0.0095	*springer*
1626831_at	10.78	9.69	2.14	0.0477	*atilla*
1624819_s_at	9.48	11.24	-3.39	0.0309	*gypsy*
1626695_at	5.83	4.81	2.02	0.0114	*CG9518*
1634993_at	6.27	5.06	2.31	0.0161	*CG40498*
1641639_at	5.71	4.24	2.77	0.0497	*esg*
1624663_a_at	4.42	6.23	-3.51	0.0438	*vis*
1627008_at	5.39	6.83	-2.70	0.032	*CG15034*
1628721_at	8.52	7.40	2.17	0.0065	*CG9864*
1630258_at	8.84	6.92	3.78	0.0013	*GstD2*
1625246_at	8.51	7.50	2.01	0.0369	*Hesr*
1624505_at	7.97	6.44	2.89	0.0356	*Lip4*
1624506_at	9.58	6.01	11.87	0.014	*Jon25Biii*
1641727_at	10.41	6.43	15.86	0.0338	*CG17470*
1626586_at	9.57	6.02	11.69	0.0449	*Obp57a*
1641390_at	7.11	8.35	-2.37	0.0376	*dpr19*
1641349_at	6.99	7.99	-2.00	0.0186	*rdgA*
1632570_at	6.26	7.30	-2.05	0.0415	*dati*
1639364_at	7.88	5.45	5.38	0.0071	*Jon44E*
1629050_at	8.03	5.00	8.14	0.0306	*CG10477*
1632584_at	6.64	5.17	2.76	0.0374	*CG3344*
1625948_at	7.11	4.44	6.35	0.0185	*CG17633*
1624137_at	6.97	4.51	5.52	0.0117	*CG11911*
1630804_at	7.33	4.43	7.49	0.0259	*Oatp33Eb*
1641190_at	8.39	4.37	16.24	0.01	*Jon65Aii*
1641412_at	7.32	5.36	3.90	0.0197	*CG31809*
1629009_at	7.85	6.44	2.65	0.0013	*Cyp28a5*
1639961_at	7.11	6.08	2.04	0.0028	*Dh44-R2*
1631164_a_at	7.09	5.92	2.25	0.0046	*Dh44-R2*
1635878_s_at	7.52	6.05	2.75	0.0094	*CG17571*
1630244_s_at	7.83	6.80	2.04	0.016	*CG31809*

D

GO molecular function	REFLIST (13757)	Ote UP (23)	expected	enrichment	FDR
serine-type peptidase activity (GO:0008236)	253	7	0.42	16.55	4.08E-04
serine hydrolase activity (GO:0017171)	254	7	0.42	16.48	2.09E-04
serine-type endopeptidase activity (GO:0004252)	231	6	0.39	15.54	1.05E-03
endopeptidase activity (GO:0004175)	384	6	0.64	9.35	1.52E-02
peptidase activity, acting on L-amino acid peptides (GO:0070011)	537	8	0.9	8.91	1.51E-03
peptidase activity (GO:0008233)	547	8	0.91	8.75	1.29E-03

Supplementary Figure 1. Loss of Chk2 rescues germarial phenotypes in *ote* mutants. **A.** (Top, left) Schematic of a germarium with GSCs (red), differentiating germ cells (orange), niche cells (gray, dashed line) and somatic cells (gray). GSCs are distinguished by spectrosomes (green circles), whereas differentiating germ cells carry fusomes (branched green). Confocal images of germaria stained for Vasa (red) and Spectrin (green). Genotypes are noted at the top of each image. Dashed lines indicate position of the GSC niche. Scale bars represent 25 µm. **B.** Quantification of the number of spectrosome-containing germ cells per germaria in less than one-day old females. Genotypes are noted below each bar and the number of germaria assessed from a minimum of five ovaries is noted above each bar. **C**. Left: Heatmap of the log2 signal intensities of probe sets that significantly changed in NULL versus HET comparisons that were highlighted in the volcano plot in Fig. 2E; unsupervised clustering reveals no segregation by genotype. Right: Table of probesets with corresponding average intensity, fold change, statistics and gene name. **D.** PANTHER test results reveal over-representation of serine proteases among the genes up-regulated in *ote*$^{-/-}$ ovaries.

Supplementary Figure 2

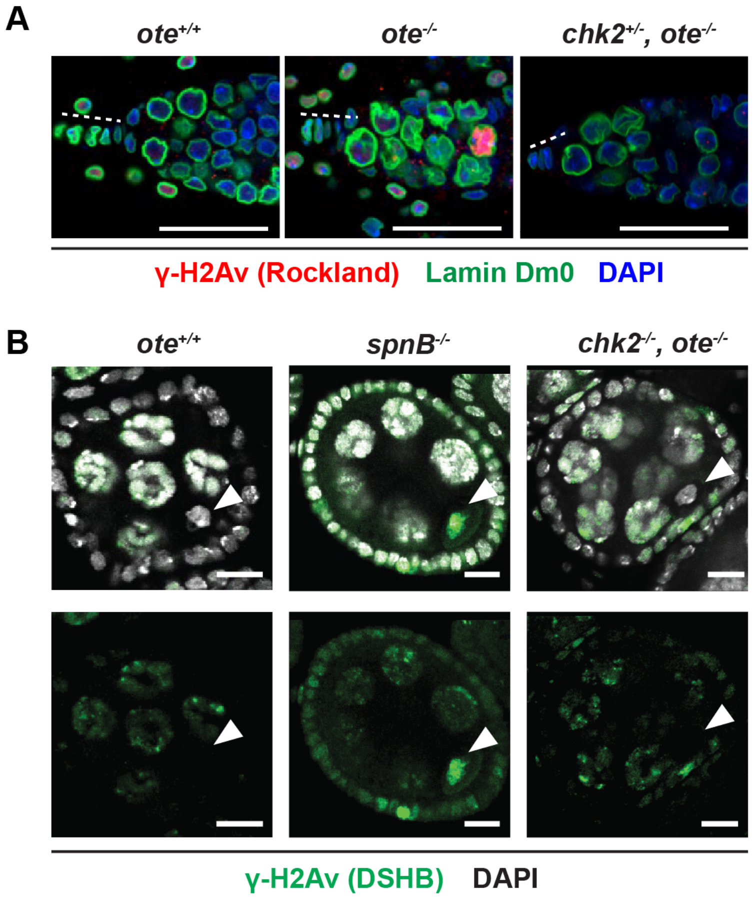

Supplementary Figure 2. Analysis of DNA damage and repair in *ote* mutants.

A. Confocal images of germaria co-stained with antibodies against Lamin Dm0 (green) and a second antibody from Rockland that recognizes that DNA damage marker γ-H2Av (red, rabbit anti-γ-H2Av, Rockland Bio), and DAPI (blue). Genotypes are noted at the top left of each image. The $chk2^{-/+}$ corresponds to $chk2^{P6}$ or $chk2^{P30}$ and $ote^{-/-}$ corresponds to $ote^{B279G/PK}$. Scale bars represent 25 μm. **B.** Confocal images of stage 5 egg chambers that carry 15 nurse cells and 1 posteriorly-positioned oocyte surrounded by a single layer of somatic cells. *Top:* egg chambers were stained with antibodies against γ-H2Av (DSHB, Green) and DAPI (blue). *Bottom*: γ-H2Av channel only. In $ote^{+/+}$ egg chambers, NC nuclei are polyploid due to multiple rounds of endoreduplicative replication cycles. As a result, these nuclei are large and carry

DSBs, evidenced by γ-H2Av staining. However, the meiotic DSBs of the oocyte nuclei are repaired in this stage egg chamber. In *spnB*$^{-/-}$ egg chambers, DSBs cannot be repaired. As a result, γ-H2Av staining is seen in somatic cells, NC and the oocyte nuclei. In *chk2, ote* double mutant egg chambers, γ-H2Av staining is seen in NCs, but absent in oocyte nuclei, evidence of normal DNA repair in this genetic background. Scale bars represent 10 μm.

Supplementary Figure 3

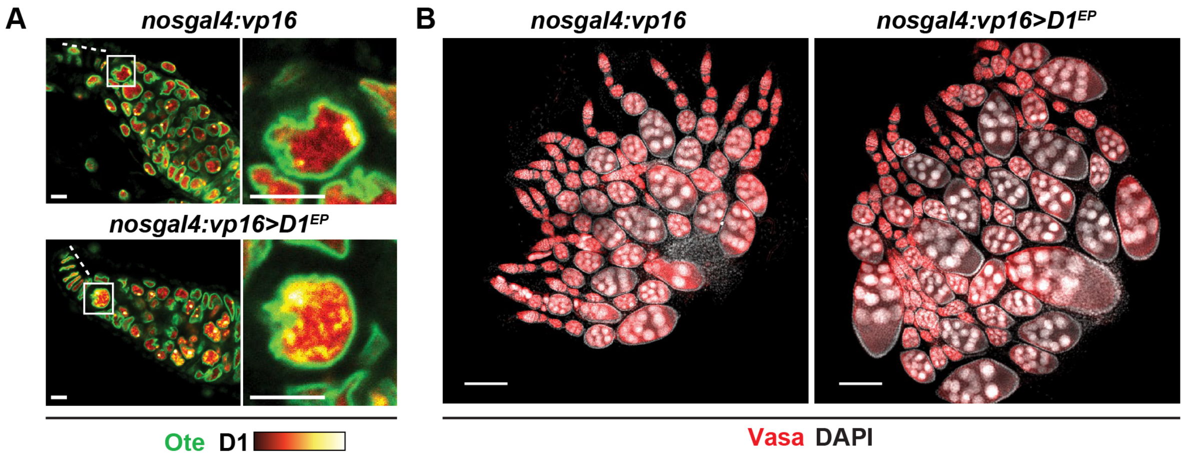

Supplementary Figure S3. Ectopic D1 production does not affect oogenesis.

A. Confocal image of germaria in *nosgal4:vp16* (driver only) or *nosgal4:vp16>D1*^{EP473} (driver and responder) females. Germaria were stained for Ote (green) and D1 (red, heatmap to show intensity level), with an enlargement of a GSC nucleus shown in the right panel. Scale bars represent 5 µm. **B.** Confocal images of ovaries dissected from less than one-day old females stained for Vasa (red) and DAPI (white). Genotypes are noted in the top of each image. Note that oogenesis is robust in females over-expressing D1. Scale bars represent 100 µm.

Supplementary Figure 4

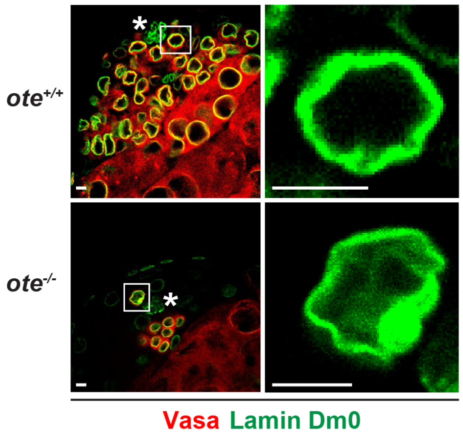

Supplementary Figure 4. Loss of Ote alters the NL in male GSCs. Confocal images of the testis stem cell niche (asterisk) in newly eclosed $ote^{+/+}$ (top) or $ote^{-/-}$ (bottom; $ote^{B279G/PK}$) animals, alongside a GSC nucleus (boxed) that shows staining for Lamin Dm0 (green) and Vasa (red), revealing nuclear structural defects in mutant male GSCs. Scale bars represent 5 μm.

Supplementary Table 1: Alleles used in study

Gene	Genotype	Nature of lesion	Source
ATM<br>*(telomere fusion)*	$w^{*};;tefu^{atm-6}, e^{1}/TM6B, Tb^{1}$	EMS mutation, W1315Stop	BL#8626 [1]
ATM<br>*(telomere fusion)*	$tefu^{atm-8}, e^{1}/TM3, Sb^{1}$	EMS mutation, L2776F, temperature sensitive	BL#8624 [1]
ATR<br>*(meiosis-41)*	$sn^{3}, mei\text{-}41^{D9}/C(1)DX, y^{1}, f^{1}$	EMS, deletion of first 163 amino acids	BL#4174
ATR<br>*(meiosis-41)*	$y^{1}, mei\text{-}41^{2}/C(1)DX, y^{1}, f^{1}$	unknown	BL#4183 [2]
claspin<br>*(cla)*	$yw; claspin^{279}/ TM6B$	C-terminal deletion of 88 amino acids, plus 25 AA	Molla-Herman [3]
claspin<br>*(cla)*	$yw, claspin^{aq4}$	C-terminal deletion of 270, plus 6 AA	Molla-Herman [3]
chk1<br>*(grapes)*	$P(PZ)\ grp^{6034}, cn^{1}/CyO; ry^{506}$	P insertion in first third of gene	BL#12219
chk1<br>*(grapes)*	$w^{1118}; PBac[RB]grp^{e00087}$ $CG33552^{e00087}$	PB insertion in first fifth of gene	BL#17813
chk2<br>*(loki, mnk)*	$y^{1}w^{67c23}; loki^{P6}/CyO$	P insertion and deletion in second exon	Y. Rong [4]
chk2<br>*(loki, mnk)*	$y^{1}w^{67c23}; loki^{P30}/CyO$	Deletion of 5'UTR and first two exons	Y. Rong [5]
chk2<br>*(loki, mnk)*	$chk2^{KD}$	Kinase-dead point mutation: D286A	T. Xie [6]
D1	$D1^{EP473}$	P{EP} insertion in the 5' UTR of the gene	[7]
otefin<br>*(ote)*	$y^{1}w^{67c23}; ote^{B279-G}/CyO, y^{+}$	PB insertion at +764	BL#16189, Geyer lab
otefin<br>*(ote)*	$y^{1}w^{67c23}; ote^{halPK}/CyO, y^{+}$	EMS mutation, R127Stop	T. Schupbach [8]
p53	$y^{1}w^{1118}; p53^{5A-1-4}$	Recombination, 3.3kb deletion of gene	BL#6815
p53	$y^{1}w^{1118}; p53^{11-1B-1}$	Recombination, Q23Stop	BL#6816 [9]
bocksbeutel<br>*(bocks)*	$y^{1}w^{1118}; bocks^{\Delta 10}$	344 bp deletion from +11	Geyer lab [10]
bocksbeutel<br>*(bocks)*	$y^{1}w^{1118}; bocks^{\Delta 66}$	728 bp deletion from +11	Geyer lab [10]

Supplementary Table 2: Primary antibodies

Protein	Source	Species	Dilution
γ-H2Av	DSHB (UNC93-5.2.1) [11]	Mouse	IFA 1:500
γ-H2AX/γ-H2Av	Rockland	Rabbit	IFA 1:500
D1	Yokiko Yamashita	Guinea pig	IFA 1:250
H3K9me3	Millipore (07-442)	Rabbit	IFA 1:100
HipHop	Y. Rong [12]	Guinea pig	IFA 1:100
HOAP	Y. Rong [12]	Guinea pig	IFA 1:100
HP1a	Covance (PRB291C)	Rabbit	IFA 1:500
lamin Dm_0	DSHB (ADL84.12)	Mouse	IFA 1:200
H3 S10ph	Millipore (06-570)	Mouse	IFA 1:200
Spectrin	DSHB (3A9)	Mouse	IFA 1:50
Vasa	Santa Cruz (sc-30210)	Rabbit	IFA 1:1,000, WB 1:5,000
Vasa	Santa Cruz (26877)	Goat	IFA 1:300
Engrailed	DSHB (4D9)	Mouse	IFA 1:50
Lamin C	DSHB (LC28.26)	Mouse	IFA 1:200
Lamin Dm0	Paul Fischer (R836)	Rabbit	IFA 1:200
Lamin Dm0	DSHB (AD:84.12)	Mouse	IFA 1:200

Supplementary Table 3: Primers used for animal genotyping

Allele	Forward Primer	Reverse Primer
$chk2^{P30}$	CTTGCTCACCTTGTTGCCATT	CAAAGGCTACAAGGGAGGGCA
grp^{6034}	TGCTGAACTTGAGCGAGAGAGCAA	ACGAGCCACACAGATACACACCAA
ote^{B279G}	ATGGCCGATGTGGACGATTTTGATTC	CGACGGGACCACCTTATGTTATTTCA
ote^{Pk}	GGATCCATGGCCGATGTGGAC	GGATCCTCAGTAGAATATGTAATAAACGCCGATTAAC
$p53^{11\text{-}1b\text{-}1}$	GTTCGCCTGGATCTTAATTA	AATCGCTGCATGCGGTAGTA
$p53^{5A\text{-}1\text{-}4}$	AGCTAATGTGACTTCGCATTGAACAAA	TCGATAAACATTGGCTACGGCGATTGT
bocks	GGCGCCTTTCTCGCTCTTGTC	TTCCCGAGATCGAGGTGTTGTCTA
cla	GCCTTACAACACTGCCTGCATCAG	GTGCCTCCTGGATCACGGCGAC

Supplementary Table 4: Primers used to measure RNAs

Gene	Forward Primer	Reverse Primer
blood	TGCCACAGTACCTGATTTCG	GATTCGCCTTTTACGTTTGC
burdock	CGGTAAAATCGCTTCATGGT	ACGTTGCATTTCCCTGTTTC
divr 2	CTTCAGCCAGCAAGGAAAAC	CTGGCAGTCGGGTGTAATTT
divr1	GGCACCACATAGACACATCG	GTGGTTTGCATAGCCAGGAT
GAPDH	CACTCGTCGGTGTTCGATGCCAAG	TCGATGACGCGGTTGGAGTAGC
gypsy6	GACAAGGGCATAACCGATACTGTGGA	AATGATTCTGTTCCGGACTTCCGTCT
Het-A	CGCGCGGAACCCATCTTCAGA	CGCCGCAGTCGTTTGGTGAGT
inv1	GTACCGTTTTTGAGCCCGTA	AACTACGTTGCCCATTCTGG
roo	CCTCTGCGTAGGCCATTTAC	AAGGCTCGATTGACCAAATG
RpL32	AAGATGACCATCCGCCCAGCATAC	ACGCACTCTGTTGTCGATACCCTTG
TART	GCCTGGGCGTATTAGTCAGATA	ACTTATGAGACGCCTCTGTTG
vasa	CATGAACTGGAGCTTGGAAGA	AGGTGCCTCCGTAAACAATAC

Supplementary References

1 Silva, E. *et al.* ATM is required for telomere maintenance and chromosome stability during Drosophila development. *Curr Biol* **14**, 1341-1347, doi:10.1016/j.cub.2004.06.056 (2004).

2 Gatti, M. Genetic control of chromosome breakage and rejoining in Drosophila melanogaster: spontaneous chromosome aberrations in X-linked mutants defective in DNA metabolism. *Proc Natl Acad Sci U S A* **76**, 1377-1381 (1979).

3 Lee, E. M. *et al.* Drosophila Claspin is required for the G2 arrest that is induced by DNA replication stress but not by DNA double-strand breaks. *DNA Repair (Amst)* **11**, 741-752, doi:10.1016/j.dnarep.2012.06.007 (2012).

4 Abdu, U., Brodsky, M. & Schupbach, T. Activation of a meiotic checkpoint during Drosophila oogenesis regulates the translation of Gurken through Chk2/Mnk. *Curr Biol* **12**, 1645-1651 (2002).

5 LaRocque, J. R., Jaklevic, B., Su, T. T. & Sekelsky, J. Drosophila ATR in double-strand break repair. *Genetics* **175**, 1023-1033, doi:10.1534/genetics.106.067330 (2007).

6 Ma, X. *et al.* DNA damage-induced CHK2 activation compromises germline stem cell self-renewal and lineage differentiation. *Development* **143**, 4312-4323, doi:10.1242/dev.141069 (2016).

7 Smith, M. B. & Weiler, K. S. Drosophila D1 overexpression induces ectopic pairing of polytene chromosomes and is deleterious to development. *Chromosoma* **119**, 287-309, doi:10.1007/s00412-010-0257-x (2010).

8 Barton, L. J., Pinto, B. S., Wallrath, L. L. & Geyer, P. K. The Drosophila nuclear lamina protein otefin is required for germline stem cell survival. *Dev Cell* **25**, 645-654, doi:S1534-5807(13)00317-1 [pii]
10.1016/j.devcel.2013.05.023 (2013).

9 Rong, Y. S. *et al.* Targeted mutagenesis by homologous recombination in D. melanogaster. *Genes Dev* **16**, 1568-1581, doi:10.1101/gad.986602 (2002).

10 Barton, L. J. *et al.* Unique and Shared Functions of Nuclear Lamina LEM Domain Proteins in Drosophila. *Genetics* **197**, 653-665, doi:10.1534/genetics.114.162941 (2014).

11 Lake, C. M., Holsclaw, J. K., Bellendir, S. P., Sekelsky, J. & Hawley, R. S. The development of a monoclonal antibody recognizing the Drosophila melanogaster phosphorylated histone H2A variant (gamma-H2AV). *G3* **3**, 1539-1543, doi:10.1534/g3.113.006833 (2013).

12 Gao, G. *et al.* HipHop interacts with HOAP and HP1 to protect Drosophila telomeres in a sequence-independent manner. *EMBO J* **29**, 819-829, doi:emboj2009394 [pii]
10.1038/emboj.2009.394 (2010).
